# Diagnostic Accuracy of Fine-Needle Aspiration Cytology (FNAC) in Thyroid Nodule Excision Cases

**DOI:** 10.7759/cureus.60600

**Published:** 2024-05-19

**Authors:** Rajab A Alzahrani, Ali G Alghamdi

**Affiliations:** 1 Otorhinolaryngology Division, Surgery Department, Faculty of Medicine, Al-Baha University, Al-Baha, SAU; 2 General surgery Division, Surgery Department, Faculty of Medicine, Al-Baha University, Al-Baha, SAU

**Keywords:** ent clinic, bethesda system, fnac, thyroidectomy, thyroid nodule

## Abstract

Introduction: Fine needle aspiration cytology (FNAC) for thyroid nodules has a high diagnostic accuracy, according to several studies worldwide. Patients who experienced preoperative FNAC had more optimal surgical treatment than others who did not perform FNAC. Therefore, achieving an accurate FNAC procedure appears to be an important tool for the proper management of thyroid nodules. We aimed to study the accuracy and challenges of the thyroid FNAC diagnostic tool in the Al-Baha region, Kingdom of Saudi Arabia.

Methods: The study involves 52 patients with thyroid nodules who underwent preoperative FNAC and postoperative histopathology with the same surgery and pathology team at Al-Baha region in 2022-2023.

Results and Conclusion: The mean age of the included patients was 47.7 years, with a female predominance. The diagnostic accuracy was 90%, and the main cause of inaccurate diagnosis was processing challenges, where the majority of cases were taken on the palpation-only technique, a few cases were ultrasound-guided, and the only technique used in the laboratory was conventional smears. The applied interrater reliability Cohen kappa coefficient (κ) for the clinical-histopathological agreement was "moderate agreement". We recommend using and evaluating more cytological techniques in addition to the currently used conventional smears in pathology laboratories to enhance the efficacy of the FNAC diagnosis of thyroid lesions.

## Introduction

Thyroid nodules can be detected in more than half of the general population, representing a diagnostic challenge for surgeons and sometimes for pathologists as well. The incidence of malignancy is around 5% of all nodular lesions of the thyroid gland [1,2]. Accurate preoperative detection of malignancy is a difficulty for clinicians dealing with thyroid nodules; consequently, fine needle aspiration cytology (FNAC) is considered the most valuable diagnostic tool to date [3,4]. It is a relatively safe, simple, and cost-effective procedure. Although it is less accurate than standard histopathological assessment, it could help avoid potentially unnecessary and invasive surgical procedures [3].

The National Cancer Institute (NCI) held the Thyroid FNAC State of the Science Conference in 2007, and its goal was to standardize diagnostic terminology, morphologic criteria, and risk of malignancy for reporting thyroid FNAC. The conference came up with a 6-tier system that they called The Bethesda System for Reporting the Thyroid Cytopathology (I = non-diagnostic, II=benign, III=atypia/follicular lesion of undetermined significance, IV = follicular neoplasm/suspicious for follicular neoplasm, V=suspicious for malignancy, and VI=malignant) [5]. However, based only on the cytological examination of thyroid nodules, some follicular lesions are difficult to distinguish malignant from non-malignant entities, particularly the follicular adenoma from carcinoma [6].

In Saudi Arabia, several studies investigated the utility of different preoperative tools for detecting thyroid malignancy, and FNAC was the most widely distributed and significant diagnostic procedure. Al-Jabr et al. reported that FNAC was a sensitive and specific initial diagnostic test for the pre-operative assessment of patients with thyroid swellings in Riyadh, Saudi Arabia. The authors also mentioned that the implementation of this standardized cytological reporting system resulted in an improved understanding of the cytological results and, subsequently, the management of nodular thyroid disease [2].

According to the previous studies, the false-negative diagnosis was caused mainly by specimen problems, while interpretation errors led to most of the false-positive diagnoses. Therefore, the initial assessment of patients should include a detailed relevant history in addition to physical and radiological examinations to increase the accuracy of the pre-operative testing [4,7,8]. This article tried to study the accuracy rate and causes of misdiagnosis of preoperative thyroid nodules’ FNAC at a referral hospital in the Al-Baha region, Kingdom of Saudi Arabia (KSA).

## Materials and methods

This is a retrospective analysis done in Prince Mishari Bin Saud Hospital-Baljurashi (PMS Hospital), a tertiary hospital in Al-Baha Region, Kingdom of Saudi Arabia, that receives patients and/or tissue samples of thyroid nodular lesions for examination and management of the eligible cases by the team of general surgery or head and neck surgery. Cytological and histological examinations of the specimens were prepared and examined by the same pathology team. The study protocol was approved (ID number: REC/SUR/BU-FM/2024/34) by the Research Ethical Committee at the Faculty of Medicine, Al-Baha University. 

Patients who underwent preoperative thyroid FNAC and fulfilled the inclusion criteria at the study hospital were identified to compare their FNAC results with the postoperative surgical pathology findings and to assess the clinico-histological agreement. The related data were retrieved from electronic medical and laboratory records, and 52 patients were included. The FNAC procedure was performed by head and neck surgeons or radiologists, either by palpation or via ultrasound guidance.

The aspirates were prepared by the cytopathology laboratory staff as direct smears (Papanicolaou stain and Geimsa stain), and cell block preparation was done when possible. All cases were initially evaluated by the pathologists according to the recommended diagnostic categories (six categories) of the Bethesda system thyroid cytopathology report, including nondiagnostic or unsatisfactory (Category 1), benign (Category 2), atypia (Category 3), suspicious for follicular neoplasm (Category 4), suspicious for malignancy (Category 5), and malignant (Category 6). Adequacy was determined by two pathologists on the basis of the standard Bethesda criteria [4].

**Figure 1:**
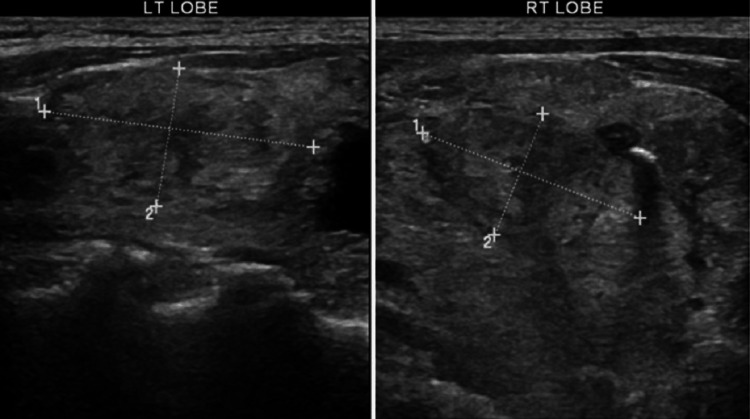
Representative ultrasound images Ultrasound images of the right and the left lobe of the thyroid showing multiple hypoechoic nodules that were proven by histopathology as nodular goiter negative for malignancy.

**Figure 2:**
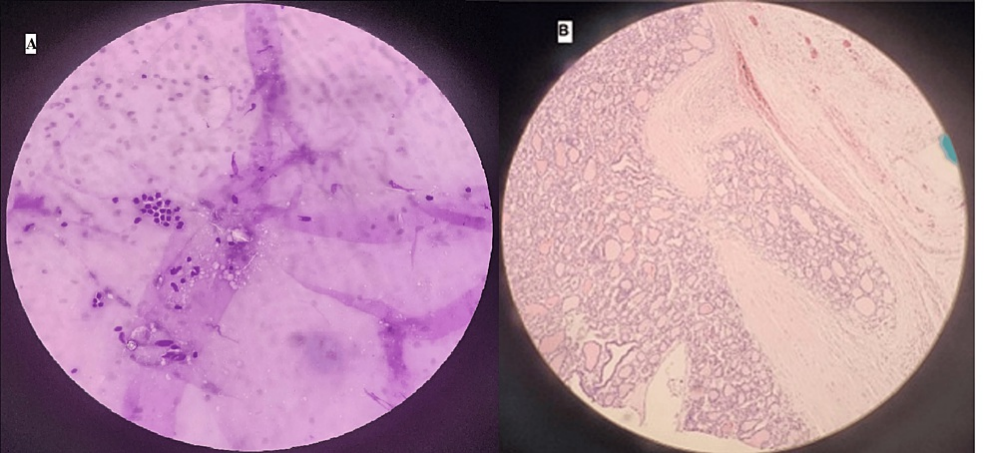
Histopathology images A case of follicular thyroid nodule; A) Preoperative FNAC showing benign features, Category 2. B) Postoperative histopathology showing follicular lesion with capsular invasion finally diagnosed as follicular carcinoma (H&E, 40x).

Inclusion criteria include patients treated with surgical excision in the Al-Baha region, preceded by the FNAC and followed by histological examination performed by the same and complete staff members at the hospital, including the nurse, specialist, and consultant of surgery (with or without the radiologist). The indications for surgical intervention included compressive symptoms, cosmetic reasons, cytological suspicion, or clear malignant cytological features. Study participants were retrieved for age, sex, recorded clinical preoperative diagnosis, relevant ultrasound findings (Figure 1), FNAC results, and postoperative final histopathological diagnoses (Figure 2).

Misdiagnosis means overdiagnoses or underdiagnoses rendered by cytopathologists. All of the slides and smears from the misdiagnosed FNACs were reexamined to determine whether the misdiagnosis was due to sampling problems or interpretation errors.

Statistical analysis

All the statistical analyses for this study were performed using an Excel sheet to produce exploratory data analysis and descriptive statistics. The Cohen coefficient effect was used in this study. The Kappa result was interpreted as follows: values ≤ 0 indicating that there was no agreement, 0.01:0.20 as none to slight, 0.21:0.40 as fair, 0.41:0.60 as moderate, 0.61:0.80 as substantial, and 0.81:1.00 as almost perfect agreement [9]. The p-value was considered significant if it was less than 0.05.

## Results

The mean age of the studied patients was 47.7 ± 11.9 (SD) with a 25-68 years range with female sex being predominant; 40/52 (77%) were female, and the rest (23%) were male patients. The thyroid nodules are multinodular in 36/52 (69.2%) of patients, and 30.8% are solitary lesions. The average nodular size was 3.13 ± 0.97 cm in maximal diameter, according to the radiological and gross pathological measurements.

Clinical diagnoses for the included cases are as follows: multinodular goiter in 30/52, neoplastic lesion in 3/52, thyrotoxicosis in 2/52, thyroid cysts in 2/52, and nodular lesion of undetermined clinical behavior in 15/52 patients. A total of 28/52 cases showed a kind of discrepancy on histopathological examination; papillary thyroid carcinoma was rendered for 13/52, follicular carcinoma in 2/52, noninvasive follicular thyroid neoplasm with papillary-like nuclear features (NIFTP) in 6/52, immunoglobulin (Ig)G4 Hashimoto thyroiditis in 1/52, incidental papillary carcinoma (0.5 cm maximal diameter of the tumor) in two patients, follicular adenoma in seven patients, nodular goiter in 19/52, and follicular variant of papillary in 2/52. The clinical-histopathological agreement percentage for the excised nodules is 61.3%. The applied Interrater reliability Cohen kappa coefficient (κ) revealed moderate agreement p (.000).

All 52 patients underwent preoperative FNAC; the post-operative histology was benign in 30/52 (58%) divided into nodular goiter in 22/30 and follicular adenoma in 8/30; however, malignant cases were 22/52 as follows: 12 papillary carcinoma (10 were pre-operative Category 5, one Category 3, and one Category 6); eight NIFTP (five Category 4, one Category 2, and two Category 3); and two follicular carcinoma (one Category 2 and one Category 3). The accuracy rate of the preoperative FNAC, according to the Bethesda system, was 90% in this study. Papillary carcinoma was the commonest malignancy on histological diagnosis, and preoperative Category 2 was the predominant category (Tables 1, 2).

**Table 1:** Cytohistological correlation for the studied cases

Category (n)	Nodular goiter	Adenoma	NIFTP	Carcinoma	Other
Category 2 (25/52)	17/25	6/25	0/25	2/25	0/25
Category 3 (12/52)	5/12	0/12	5/12	2/12	0/12
Category 4 (8/52)	0/8	2/8	5/8	1/8	0/8
Category 5 (6/52)	0/6	0/6	1/6	5/6	0/6
Category 6 (1/52)	0/1	0/1	0/1	1/1	0/1

**Table 2:** Analysis of the discrepancy cases

Under-diagnosis	Number	Cause	Variant
Thy2: cancer	2/52	Capsular invasion	FA: follicular carcinoma
Thy2: NIFTP	0	NA	NA
Thy3: cancer	2/52	Processing	Papillary carcinoma
Thy3: NIFTP	5/52	NA	NA
Thy4: cancer	1/52	Interpretation	Papillary carcinoma
Over-diagnosis	Number	Cause	Variant
Thy6: Benign	0	NA	NA
Thy5: Benign	0	NA	NA
Thy5: NIFTP	1/52	Processing	NA
Thy4: Goiter	0	NA	NA

The diagnostic accuracy rate of the preoperative FNAC, according to the Bethesda system in this study, was 90%. All cases were prepared in the laboratory department as conventional smears. Surgical excision was provided for the studied cases; 25/52 experienced total thyroidectomy, 20 hemithyroidectomy, five cases were excised as hemithyroidectomy, then completion was done, and two patients were managed by lobectomy.

## Discussion

Thyroid cancer (papillary and follicular) is the most prevalent endocrine malignancy among the female population in Saudi Arabia and the ninth most common in the Saudi male population [10]. Its incidence has been increasing in the Kingdom over the past few years. However, the etiology of this endocrine cancer is still not clear, and an accurate diagnosis is crucial for proper management [10,11]. Optimal use of laboratory and biochemical tests to diagnose and evaluate patients with thyroid nodules or thyroid-different malignant lesions is limited and requires studying and an appreciation of the pathophysiology and the factors implicated in both thyroid hyperplasia and neoplasia (especially thyrotropin, TSH), activating mutations of the TSH receptor, and the oncogenic transformations [12,13]. Routine preoperative thyroid function tests are usually requested for patients preparing for surgery to achieve an euthyroid state not for detecting the thyroid nodular malignancy [14].

In patients with thyroid nodules, FNAC plays a crucial role in the initial evaluation and establishment of treatment strategies; however, a number of malignant cases might be missed, in addition to the relatively high rate of inadequate or unsatisfactory samples, which necessitates repeat testing [15,16]. Additionally, instances of false-positive malignancy diagnoses may occur, resulting in needless thyroid surgery that carries a long-term postoperative morbidity risk ranging from 2% to 10%. The FNAC results have a substantial impact on the decision to pursue surgery rather than conservative management; therefore, a consistent reporting process and rigorous evaluation of the diagnostic utility of thyroid FNAC are required [16,17].

The diagnostic accuracy rate of the preoperative FNAC, according to the Bethesda system in this study, was 90%. This rate is lower than what was calculated by several studies. A recent study on patients from Somalia recorded an overall sensitivity, specificity, and accuracy rate of cytological-histological correlation of 91.1%, 96.6%, and 94.9%, respectively [18], and the study conducted in 2010 on patients who underwent thyroidectomy recorded an accuracy rate of FNAC of 94.5% and a specificity of 97.3% [19], which is higher than what we report in this study. This may be due to the application of ultrasound guidance for aspiration or using different cytological techniques affecting the accuracy and processing problems. 

The collection and processing of thyroid cytological specimens are crucial to maintaining the integrity of thyroid FNAC. In general practice, conventional smears are prepared from aspirate material from most of the anatomical sites of the human body. Following the 1990 Federal Drug Administration approval of ThinPrep for processing non-gynecologic cytological material, a growing number of pathology laboratories process FNAC thyroid rinse by this method [20]. ThinPrep has many advantages compared with conventional smears; it is clearer and easier to read due to minimizing obscuring blood and mucous, providing higher accuracy in the assessment of the cells and the ability for ancillary testing [21,22]. Some authors suggested that ThinPrep is diagnostically superior to conventional techniques in certain non-gynecologic specimens, including thyroid nodules; however, several studies have shown that it has diagnostic equivalence to conventional techniques but acknowledges cytologic differences according to the amount of colloid, background, and nuclear details [20,23,24]. Due to the artifacts resulting from ThinPrep in thyroid aspirates, some authors have established that pathology laboratories should not use ThinPrep as the sole method for FNAC material preparation in thyroid samples [25-27].

Another cytologically new technique is liquid-based cytology, which is an automated technique (machine-based) that yields a single Pap-stained smear with a circular, evenly spread material in the center of the slide. It gives an advantage to a clean background for the monolayered cells with well-preserved morphology and consumes less screening time. Liquid-based cytology is widely used in gynecological smears nowadays; however, a few studies have been reported in the literature for the interpretation of thyroid nodules [28].

Some previous studies concluded that liquid-based cytology serves as a useful adjunct diagnostic method, and its combination with conventional smears has been shown to reduce unnecessary thyroidectomies due to the good identification of malignant and suspicious thyroid lesions [28,29]. However, a recently published meta-analysis study by Kang et al. in 2024 [30] concluded that the diagnostic accuracy of FNAC in thyroid nodules did not significantly differ among conventional and/or liquid-based cytology, but comparing two kits (ThinPrep and SurePath) revealed a significant difference, suggesting that SurePath kits might be more accurate.

In this study, the majority of misdiagnosis cases were referred to as processing errors and laboratory staff used only conventional techniques, recommending consideration and evaluation of other processing techniques. The small number of the studied cases in addition to lack of comparing the US-guided with the palpation only sampling are the main limitations of this study.

## Conclusions

Laboratory investigations and clinical examination have a limited role in detecting the nature of thyroid nodules exactly, except the FNAC tool has been proven to be the best pro-operative diagnostic tool to present. This study showed a moderate conformity between pre-operative clinical and post-operative histopathological thyroid examinations. FNAC achieved a total accuracy rate of 90% in the study hospital in Saudi Arabia, and the majority of misdiagnoses were due to processing issues. We recommend using additional cytological techniques in addition to the currently used conventional smears at the pathology laboratories to enhance the efficacy of the FNAC diagnosis of thyroid lesions. More studies and audits on the efficacy of palpation only versus guided techniques for thyroid FNAC are also recommended.
